# NAMs for closing knowledge gaps in assessment of respiratory uptake – a PARC project

**DOI:** 10.3389/ftox.2026.1764276

**Published:** 2026-06-03

**Authors:** Pierre-André Billat, Tanja Hansen, Véronique De Bruijn, Rayane Boufalaas, Simone Stefano, Anita Sosnowska, Yvonne Kohl, Sandra Verstraelen, Laura Maria Azzurra Camassa, Max Spänig, Catherine Gabriel, Dimosthenis Sarigiannis, Kerstin Krätschmer, Giuseppa Raitano, Natalia Bulawska, Aude Ratier, Shan Zienolddiny-Narui, Aline Chary, Tommaso Serchi, Susana Viegas, Steen Mollerup, Sylvia E. Escher, Yvonne C. M. Staal

**Affiliations:** 1 INERIS, Experimental toxicology and modeling unit (TEAM), Verneuil en Halatte, France; 2 ITEM, Fraunhofer Institute for Toxicology and Experimental Medicine, Hannover, Germany; 3 RIVM, Bilthoven, Netherlands; 4 CNRS, Biomechanics and Bioengineering, Centre de recherche Royallieu, University of technology of Compiegne Royallieu, Compiegne Cedex, France; 5 Laboratory of Environmental Chemistry and Toxicology, Department of Environmental Health Sciences, Istituto di Ricerche Farmacologiche Mario Negri IRCCS, Milano, Italy; 6 Laboratory of Environmental Chemoinformatics, Faculty of Chemistry, University of Gdansk, Gdansk, Poland; 7 Fraunhofer Institute for Biomedical Engineering IBMT, Sulzbach, Germany; 8 Flemish Institute for Technological Research (VITO), Environmental Intelligence Unit, Mol, Belgium; 9 STAMI, National Institute of Occupational Health, Oslo, Norway; 10 Environmental Engineering Laboratory, Department of Chemical Engineering, Aristotle University of Thessaloniki (AUTH), Thessaloniki, Greece; 11 HERACLES Research Center on the Exposome and Health, Center for Interdisciplinary Research and Innovation, Balkan Center, Thessaloniki, Greece; 12 Wageningen Food Safety Research (WFSR), Part of Wageningen University & Research, Wageningen, Netherlands; 13 PériTox Laboratory, UMR-I 01 INERIS, Université de Picardie Jules Verne, Amiens, France; 14 LIST, Luxembourg Institute of Science and Technology, Belvaux, Luxembourg; 15 NOVA National School of Public Health, Public Health Research Centre, Comprehensive Health Research Center, CHRC, REAL, CCAL, NOVA University Lisbon, Lisbon, Portugal

**Keywords:** *in vitro* lung barrier models, respiratory uptake, ADME models, read-across (RAx), physiologically-based kinetic (PBK) modelling, *in vitro* to *in vivo* extrapolation (IVIVE), next generation risk assessment (NGRA), new approach methodologies (NAMs)

## Abstract

Inhalation is a major route of chemical exposure for both consumers and workers. Physiologically-based kinetic (PBK) modeling is a promising tool to understand the absorption, distribution, metabolism, and excretion (ADME) of inhaled chemicals and to predict systemic concentrations of chemicals in humans. New Approach Methodologies (NAMs) can help generate essential input parameters for PBK models. However, validated NAM-based test methods to assess uptake of inhaled chemicals are currently lacking. Reliable information on respiratory uptake is required to determine relevant exposure concentrations for evaluation of systemic effects using NAMs. This manuscript describes a project that aims to apply robust and reliable *in vitro* models to study cellular uptake, intracellular accumulation, absorption and systemic exposure of chemicals following inhalation. To evaluate the robustness and predictivity of different NAM-based barrier models, to examine appropriate *in vitro* to *in vivo* scaling strategies, and to assess sensitivity and uncertainty in the resulting PBK models, the project will focus on relatively data-rich chemicals, specifically per- and polyfluoroalkyl substances (PFAS). While some have been widely explored and others remain data-poor, the entire chemical family is of interest due to its health hazards. Therefore, the work combines experimental and modeling approaches by generating *in vitro* data on the respiratory uptake and benchmark this to existing human *in vivo* data, developing biokinetic models to better understand chemical fate within the test systems, and refining inhalation PBK models to improve estimates of systemic uptake. Read-Across (RAx) will be employed as data gap filling technique to infer on the apparent permeability of non-tested PFAS. Together, *the in vitro* and *in silico* results will inform and parameterize PBK models, ultimately enabling more reliable predictions of systemic availability. The project will deliver a workflow to combine *in vitro* and *in silico* methods to assess the uptake of inhaled substances, that could be modified and applied to other inhaled substances. Standardized *in vitro* models for respiratory uptake will improve the evaluation of inhalation as a route of exposure contributing to systemic effects, which is a key requirement for quantitative *in vitro* to *in vivo* extrapolation (qIVIVE) and supports the implementation of next-generation risk assessment (NGRA).

## 1 Introduction

Daily exposure to airborne substances, like, e.g., environmental pollutants, can lead to toxicological effects in the respiratory tract (Sofia et al., 2025). In addition, inhaled substances may be absorbed, enter the blood circulation and subsequently exert systemic toxicological effects in other human organs (Orru et al., 2025). Beyond exposure of the general population to, e.g., household products and environmental pollutants, understanding inhalation uptake is particularly relevant for occupational settings, where workers are often exposed to airborne substances at relatively high concentrations.

In inhalation toxicology, dosimetry refers to the quantitative characterization of the amount of a substance interacting with its biological target and operates at two distinct levels. *In vivo*, dosimetry encompasses regional deposition, mucociliary clearance, and dissolution of inhaled substances in the respiratory tract, processes governed by aerodynamic diameter, airway architecture, and airflow dynamics. New Approach Methodologies (NAMs) are available to predict local deposition of, e.g., aerosols and particles in the lungs (Ramanarayanan et al., 2022). *In vitro*, dosimetry describes the fraction of the actual amount of substance that effectively reaches the apical cell surface, which drives both the cellular dose and the concentration for transepithelial transport. Accurate *in vitro* dosimetry is therefore a prerequisite for calculating meaningful apparent permeability (P_app_) and for scaling *in vitro* data to *in vivo* conditions within physiologically-based kinetic (PBK) models. In the present work, the focus is entirely on *in vitro* dosimetry: factors such as protein binding, plastic adsorption, and evaporative loss that reduce the fraction of applied substance reaching the cells are explicitly modelled, while *in vivo* respiratory tract deposition modelling is outside the scope of this project. Details are described in Table 1.

**TABLE 1:** Parameters impacting dosimetry and hazard assessment. These include physico chemical properties, including particle/aerosol characteristics, features of the exposure system and properties of the respiratory epithelium. All of these may impact *in vivo* and *in vitro* dosimetry, and thus (q)IVIVE. The table gives an overview on how these are addressed in the project described in this manuscript.

Parameter group	Parameter	Impact *in vivo* respiratory dosimetry	Impact *in vitro* exposure systems	Implementation/limitation/mitigation
Physicochemical properties	Solubility	Influences dissolution rates and transfer to epithelial lining fluid	Determines bioavailable fraction at cell surface	Poorly soluble chemicals will be dissolved in DMSO but not exceeding 0.1% V/V of the final formulation
	Volatility	Influences partitioning between air and blood	Affects actual dose (potential losses); temperature dependent	Effective concentration will be measured at different timepoints and at the last timepoint it will be confirmed whether the mass balance can be retained
	Lipophilicity (logP)	Influences permeability, distribution and toxicity	Affects actual dose exposure (binding on plastic/proteins), and cellular accumulation	Used for *in silico* predictions (QSAR, QSPR, biokinetic and PBK models)
	Ionisation (pKa)	Influences permeability	Affects transport across epithelial barrier	Considered qualitatively based on the pH of the barrier/basal medium
	Hygroscopicity	Influences deposition pattern	Affects deposition and bioavailability	Impact on exposure vs. dose relationship
Physicochemical properties specifically for particles/aerosols	Particle size distribution	Determines Mass Median Aerodynamic Diameter (MMAD) and thereby deposition, mechanisms to influence initial deposition location, fraction and subsequent transport mechanisms	Affects impaction, sedimentation, diffusion, and deposited dose	Initially not applicable for our project, as quasi-ALI exposure is used
	Particle density	Determines MMAD and thereby deposition, mechanisms to influence initial deposition location, fraction and subsequent transport mechanisms	Affects impaction, sedimentation and deposited dose	Not applicable for our project
Exposure conditions	Air flow rate (when using airborne exposure methods)	Controls rate of deposition/absorption	Controls rate of deposition/absorption	Defined and controlled experimentally
	Exposure duration	Determines cumulative delivered dose	Directly affects uptake kinetics and permeability	Integrated in experimental design and biokinetic/PBK simulations to run or to extrapolate to realistic exposure conditions
	Exposure concentration	Determines cumulative delivered dose and nature of toxicity	Important for concentration dependent uptake (i.e., saturation of uptake processes)	Concentrations will be measured in the different matrices (cell culture media, cell lysates, apical washes and insert membranes), different dose levels will be tested to evaluate impact on absorption linearity
	Free vs. bound fraction	Controls bioavailable concentration	Major determinant in *vitro* systems	Considered using biokinetic models or experimentally determined values
Respiratory epithelium	Surface area	Varies with on age, sex, race, disease stage; impacts absorption	Determines extrapolation to whole lung	Used for scaling to PBK models (*in vitro/in vivo* extrapolation)
	Barrier integrity, paracellular permeability	Reduces permeability to chemicals/particles	Quality control (QC) for permeability assays	Used as QC
	Transporter expression/activity	Drives active transport	Influences uptake and scaling	Currently limited data available, proteomics analysis of the cell models may provide information
	Specific enzyme and associated metabolic activity	Metabolism of parent compound into active/inactive compounds	Affects effective dose and kinetics, impacts on toxicity	Poorly characterized, identified as limitation. However, PFAS are poorly metabolized
	Mucociliary clearance	Removes particles from airways	Not reproduced in static systems, may be mimicked with washing	Not addressed
	Translocation across epithelium	Governs systemic uptake and affects local concentration	Central endpoint of *in vitro* permeability models	Directly measured (papp)
	Cell type, morphology, thickness	Dependent on the location in the respiratory tract	Properties such as cilia or mucus may affect the availability and free concentration	Different cell models of alveolar and bronchial region included

PBK models estimate plasma and tissue-specific concentration-time profiles within the body from an external exposure. However, their predictive reliability depends heavily on accurate input data for chemical uptake predictions (Dallas et al., 1994). A critical gap remains in the quantitative characterization of transepithelial permeability across the respiratory epithelium, which directly governs systemic uptake following inhalation. Addressing this process is the central objective of the present PARC project. Compound-specific information on permeability across the lung epithelial barrier can be derived from NAMs, including different approaches such as *in silico* predictions based on physico-chemical properties and *in vitro* models (Medinsky and Bond, 2001; Punt et al., 2022; Nowak et al., 2025). *In vitro* barrier models of the respiratory tract, specifically those that are cultured at the Air-Liquid Interface (ALI) can provide human-relevant and substance-specific information on respiratory transepithelial transport (Panduga et al., 2017; Sakagami, 2020; Huang et al., 2024).

Standardization and advancing *in vitro* approaches for assessing respiratory uptake will improve the evaluation of inhalation as a contributor to systemic effects. Establishing *in vitro* systems that form a tight and physiologically relevant respiratory barrier is essential for applying NAMs in the regulatory risk assessment of airborne substances, including under frameworks such as REACH and OSH, while supporting adherence to the 3Rs principles (replacement, reduction and refinement of animal testing). Reliable information on respiratory uptake is needed to quantify inhalation absorption more accurately. To integrate *in vitro* permeability data into a PBK model and predict systemic concentrations, a scaling step is needed. Scaling factors should at least account for the differences between the surface of the *in vitro* test system and the whole lung, and potentially for variations in transporter and metabolic enzymes expression or activity. Transporter expression and metabolic activity (for metabolized compounds) in lung barrier models remain poorly characterized while improved characterization would help to refine PBK parameterization, thereby strengthening the link between *in vitro* approaches and systemic exposure predictions.

Reliable data on respiratory uptake are critical for next-generation risk assessment (NGRA), as this provides information on the concentration of a substance reaching systemic circulation and potentially affects target organs. For substances unable to cross the respiratory barrier, effects are expected to remain at the site of contact (local effects). As such, understanding respiratory transepithelial permeability determines whether the inhalation route contributes meaningfully to aggregated exposure across different exposure scenarios, including occupational and consumer settings. Additionally, information on the relevance of the inhalation route of exposure can support interpretation of hazard data generated under other exposure routes under REACH, potentially avoiding additional animal studies conducted via alternative routes. With the increasing use of NAMs, information on systemic concentrations is required to decide if further hazard assessment targeting systemic organs is needed.

The development and validation of innovative *in vitro* and *in silico* approaches, such as permeability prediction via Read-Across (RAx) based on physicochemical descriptors, biokinetic models to support and refine *in vitro e*xperiments, and PBK simulations, to study respiratory uptake will make a significant contribution to the shift away from animal testing through NAMs (Pandey and Roy, 2025; Sadekar et al., 2025). In addition, focusing on a class of substances enables the exploration of relationships between physicochemical properties to the reliability of the respiratory uptake data and use of *in vitro* models. With the experience of a single class of compounds, the combination of methods can efficiently be applied to other classes of chemicals, provided that compound-specific data and validation against human-relevant benchmarks are available. Moreover, the diversity of airborne compounds, being inhaled as either gases, vapors, liquid or particulate aerosols requires guidance on the use of optimal *in vitro* exposure methods to assess respiratory uptake (Staal et al., 2024a). To assess respiratory uptake, we need (1) standardized and robust NAM-based respiratory transepithelial permeability methods, (2) scaling strategies to use *in vitro* permeability information into PBK models.

The aim of the project described in this manuscript, (RESUPT, PARC task 5.3.4b) is to develop and assess suitable *in vitro* barrier models to better characterize the respiratory permeability of inhaled chemicals. The focus on inhaled chemicals excludes particles, as there uptake mechanisms are different from chemical uptake, and reactive substances, as these generally cause local effects. The group of PFAS substances has been selected for this purpose, as PFAS are structurally related and contains both volatile and non-volatile compounds (vapor pressure between 2.48 × 10^−6^ for PFOS to 0.15 mmHg for PFOA). Their structural similarity also provides the opportunity to apply RAx methods to derive apparent permeability (P_app_), for data-poor PFAS. In parallel, collection of information on transporters expressed in *vitro* lung barrier models, and comparison with information on expression of these transporters in the human respiratory tract will aid the selection of choosing relevant *in vitro* barrier models to assess respiratory uptake and scaling *in vitro* parameters to use in PBK models. Data will provide input for PBK development and modelling, including integration into existing models (Nowak et al., 2025).

Rather than providing a comprehensive review of respiratory uptake of all inhaled chemicals, this manuscript uses PFAS as a case study to develop, illustrate, and validate our proposed framework for filling gaps in inhalation exposure. The proposed framework can, in principle, be applied to other non-volatile, non-reactive compounds, acknowledging that aerosols, reactive gases, and volatile organic compounds would require different dosimetry models and adapted RAx strategies beyond the scope of this work.

## 2 Approach

**FIGURE 1:**
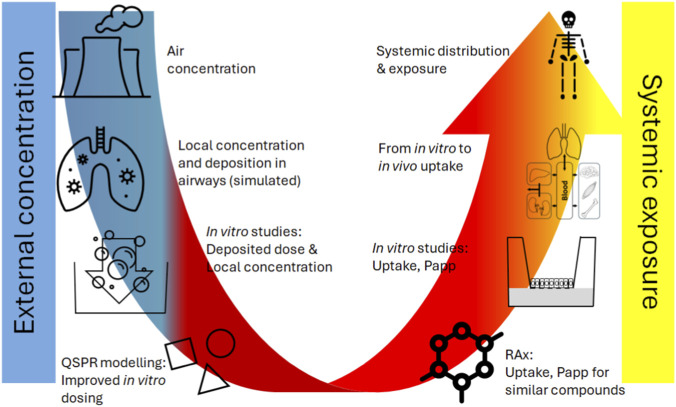
Visual representation of the project plan, adapted from Staal et al. (2024a), Airborne substances are inhaled and can deposit in the respiratory tract, leading to local exposure. To assess whether these exposures result in systemic exposures and effect, *in vitro* dosing and permeability assessments are required. This project focuses on the red part of the arrow, starting at the point of dosing, to ensure that the actual dose can be determined from the applied dose (nominal dose) and that this is relevant for human exposure. QSPR modelling will improve the determination of the actual dose, especially for compounds that have high affinity for lipids or are volatile. RAx will extend information on permeability for related substances with available *in vitro* data (see section In silico approaches to fill data gaps). *In vitro* models (see section Cell models, characterization and their transporters) will be used to determine uptake and permeability (in the cell and across the cell layer, respectively) of a selected set of substances, that will be input for PBK modelling to predict systemic distribution of inhaled substances.

The project described in this manuscript aims to provide integrated approaches, combining *in vitro* and *in silico* models to fill a knowledge gap regarding NAMs for the assessment of toxicity after exposure by inhalation. The workflow, consisting of an experimental design, parameterization of biokinetic/PBK models and scaling strategies, may serve as a transferable basis for other chemical classes provided that compound-specific data are generated and appropriate validation is performed. A visual representation of the project plan is shown in Figure 1. Information on respiratory transepithelial permeability is essential for quantifying the bioavailability of compounds in plasma as well as tissues and can influence the selection of relevant NAMs to address potential systemic effects. Guidance on the methods of exposure, applicability of barrier models, and data requirements for PBK modelling are needed to obtain relevant and reliable data.

To this end, the project consists of the following parts:

1. Define criteria for *in vitro* respiratory barrier models to evaluate their usefulness in assessing respiratory uptake. Aspects to be considered are i) the ability to form a tight barrier and produce reproducible data ii) the *in vitro* to *in vivo* comparisons of transporters and enzymes, iii) the knowledge and methods used to account for loss of compounds based on the *in vitro* set up (like adsorption to plastics, evaporation, etc.).
2. Generate experimental data on the *in vitro* biokinetics of a set of substances from the same class to define which *in silico* models and experimental approaches (suitable dosing methods) are needed to assess the loss of substances due to the *in vitro* experimental design. Provide guidance for appropriate and human relevant exposure methods for *in vitro* models. An assessment of non-specific binding to experimental devices and biological matrices will be performed using dedicated experimental assays. In cases where parameters are not readily determinable, Quantitative Structure Activity Relationship (QSAR), Quantitative Structure Property Relationship (QSPR) models will be used to facilitate their estimation. This original data can help improve the inhalation permeability models currently under development by highlighting their weaknesses and strengths. In addition to characterising the in vitro models, the results will be used to predict the cellular uptake, intracellular accumulation and absorption of chemicals, after exposure within the tissue of interest (bronchial or alveolar). With this, we will better understand the permeability of the lung in the experimental devices and enable the use of NAMs in hazard and risk assessments to reduce and refine experimental approaches.

3. Further develop *in silico* tools to parametrize inhalation PBK models (Nowak et al., 2025), including interaction with proteins that affect bioavailability and distribution. The PBKiT model is a PBK model that addresses the lung as a multi-compartment model, with three regions (tracheobronchial, bronchiolar and alveolar) and 25 sections, reflecting their specific physiology and mechanistic processes such as airflow, permeability and clearance. Mucociliary clearance and phagocytosis are included, and aerosol deposition is predicted by the Multiple-Path Particle Dosimetry (MPPD) model. The results of this project will provide guidance on how to use *in vitro* models of the respiratory tract, alone or in combination with *in silico* tools, to assess respiratory uptake and estimate systemic concentrations.
4. Assess the sensitivity different *in vitro* methods to define input parameters for PBK models and quantify the remaining uncertainties. Develop guidance on a tiered assessment strategy for assessing the uptake of inhaled substances. Where possible, provide insights in physicochemical properties that are indicative for local and systemic effects.

Deposition of inhaled substances in the respiratory tract depends on the physical chemical properties of the substance in the air and results in regional differences in delivered dose. (see Table 1) Such differences are important determinants not only of where effects occur, but are also of the magnitude of local and systemic exposure, since transepithelial permeability differs between the tracheobronchial and alveolar regions. Although *in vivo* deposition modelling is out of the scope of this project, the following considerations are addressed:

- *In vitro* models of both the alveolar and bronchial region of the lungs, as the two principal compartments relevant to absorption of inhaled compounds, have been included, allowing comparison of region epithelial permeability. The inclusion of models of other regions of the respiratory tract, especially the nasal regions, may be valuable to assess at a later stage, when our approach of using *in vitro* and *in silico* tools indicates to be promising.
- While *in vivo* respiratory deposition modelling, such as MPPD, is not included, *in vitro* deposition modelling is. This is an important aspect for both toxicity and uptake assessment, as not all applied substances may actually reach the cells. Factors such as protein or lipid binding, plastic binding, or evaporation may reduce the dose actually reaching the cells. Such properties can be modelled with QSPR modelling and integrated into a biokinetic model. More precise dose estimation enhances the determination of uptake of a substance and calculation of the P_app_.

The chapters below summarize ALI-cultured cell models and their properties that are important for uptake assessment, the selection of chemicals, the use of QSPR and QSAR models in respiratory uptake assessment, and the use of these data for PBK modelling.

## 3 Cell models, characterization and their transporters

Several ALI models of the respiratory epithelium have been developed and could be applied to determine respiratory uptake. These models differ in their origin and characteristics. Important aspects of *in vitro* respiratory models for uptake assessment include the formation of a functional barrier and the ability to be cultured at the ALI, to allow airborne exposures. Other properties, like the origin of the tissue, the location of the respiratory tract and the production of surfactant or mucus, could be important for permeability assessment, but currently data are too limited to determine whether these properties are essential.

Furthermore, the expression of metabolic enzymes and transporters on the cell membrane may also affect the respiratory uptake, depending on the substance. The ideal model for *in vivo* respiratory uptake assessment should reflect the human *in vivo* respiratory permeability in the systemic circulation after inhalation. This includes, but not limited to, a continuous epithelium with tight junctions, similar uptake rates and relevant expression of transporters and metabolic enzymes, and, where applicable *in vivo* human regional differences. The chosen cell system should represent the human *in vivo* respiratory barrier. Furthermore, *in vitro* models for permeability assessment should be reliable and reproducible in obtaining uptake parameters. When multiple models meet these criteria (within a chemical applicability domain), preference is given to models that are easier to culture and less expensive, facilitating their integrations into high throughput screening.

For the purpose of this project, cell models of the respiratory tract epithelium have been selected for assessment of their applicability to assess respiratory uptake, these are described in more detail in Table 2. This section does not aim to provide a complete overview of all *in vitro* models available, but to summarize the most promising models for assessing transepithelial permeability in the tracheobronchial and alveolar regions of the lungs.

**TABLE 2:** Overview of cell models and their properties.

Cell model	Primary bronchial cells	Calu-3	HBEC2-KT, HBEC3-KT, HBEC12-KT	hAELVi	hiPSC based AEC2	ALIsens
Origin	Bronchial epithelium	Bronchial adenocarcinoma	Immortalized bronchial epithelium	Immortalized alveolar epithelium	Differentiated from human pluripotent stem cells	Immortalized cell lines, co-culture
Location respiratory tract	Bronchial	Bronchial	Bronchial	Alveolar	Alveolar	Alveolar
Primary or immortalized	Primary	Immortalized	Immortalized	Immortalized	Immortalized	Immortalized
Mucus production (y/n)^a^	yes	yes	yes	no	no	no
Surfactant production	no	no	No	yes	yes	yes
Barrier formation	yes	yes	Yes, evidence after long differentiation in ALI	yes	Yes, compared to A549	Poorly characterized, absence of functional tight junctions
Expression of metabolizing enzymes	Partially characterized	Partially characterized	Partially characterized	Poorly characterized	Poorly characterized	Poorly characterized
Expression of transporters	Partially characterized; differences based on location in lung	Partially characterized	Partially characterized	Poorly characterized	Partially characterized	Not characterized
Main limitations	Donor-dependent genetic variability and phenotypes, complex culture/handling, costly	Low or no CYP activity, variable transporter expression No physiological tight junction impacting transcellular permeability	Donor-dependent genetic variability and phenotypes, culture-dependent differentiation, limited metabolism, costly	Low or no CYP and transporter activity Very strong tight junctions, costly	Donor-dependent genetic variability and phenotypes, maturity, aging	Complex cell co-culture, unknown barrier properties, costly

^a^ alveolar epithelium does not secrete mucus but surfactant.

Our selection focusses on cells cultured on inserts as these allow different methods of exposure to airborne substances. However, in addition to these static models, the organ-on-chip models, especially those that mimic breathing function, could provide valuable input for uptake assessment as well. Lung-on-chip systems have been developed and tested to evaluate permeability by replicating the airway-capillary or alveolar-capillary interface. These systems employ a co-culture of lung epithelial cells (primary cells or cell lines) and endothelial cells, maintained under ALI conditions. Dynamics cyclic strain is applied to simulate breathing, providing mechanical stimulation that enhances barrier integrity and promotes physiologically relevant cellular phenotypes. As a result, permeability measurements obtained from these systems closely mimic those observed in *vivo* lung tissue (Zamprogno et al., 2021; Geiger et al., 2025; Liu et al., 2025).

The described cell models are suitable for exposure via different methods to aerosols, vapors, and gases, as well as to liquids applied either to the apical surface (e.g., by pipetting a small volume -max 50 µL for a 12-well insert-; quasi-ALI), aerosolized, or added to the basolateral medium to simulate systemic exposure. Importantly, the exposure mode either LLI or ALI influences both barrier integrity and uptake behavior in respiratory epithelial models, with LLI conditions yielding lower transepithelial electrical resistance (TEER) values and being less differentiated, while ALI conditions promote mucociliary differentiation, more physiologically representative tight junction architecture, and altered particle uptake kinetics. ALI exposure strongly influences barrier properties and uptake behavior while appearing to provide more relevant input for inhalation toxicology (Lacroix et al., 2018; Kaur et al., 2022; Silva et al., 2023).

The predictive reliability of a cell model for uptake assessment depends not only on the properties of the cells (barrier tightness, transporter expression and metabolic activity) but also on an appropriate characterization of the barrier before the assay and the exposure system (controls for barrier quality using TEER or other permeability markers, absence of cytotoxicity). In addition, a reference substance, ideally one for which human *in vivo* kinetic profile after inhalation is known, will be used as a comparator. To minimize experimental artefacts, it is crucial to accurately determine the applied dose (to allow calculation of mass balance) and chemical properties.

Exposures can be performed under either static or dynamic conditions, depending on the experimental design and the compound of interest. For comparison of cell models, exposures will be performed using a direct apical dosing approach under quasi ALI conditions, in which a defined liquid volume containing the same quantities of test compound for all cell models is applied to the apical surface of the epithelial layer. This approach was intentionally selected as a first, controlled step to minimize variability associated with aerosol generation and delivery, and to ensure a well-defined and reproducible applied dose across different cell models. We acknowledge that our dosing strategy is not intended to replicate realistic inhalation exposure conditions. Rather, it represents a controlled experimental setup to compare intrinsic transepithelial transport properties across models. More physiologically relevant exposure systems (aerosol exposure under dynamic ALI conditions) will be considered in later stages of the project, once suitable cell models have been identified. The actual choice of exposure system is substance-dependent, particularly for compounds with differing volatility or aerosolization potential.

## 4 Bronchial cell models

### 4.1 Primary bronchial epithelial cells

#### 4.1.1 ALI culture

Primary cells are widely used within *in vitro* inhalation toxicology. They can be obtained from different regions of the donor’s lung and can be purchased commercially or obtained during surgery, e.g., lung cancer or lung volume reduction surgery (Yaghi et al., 2010). Primary bronchial epithelial cells (PBECs, also called normal human bronchial epithelial cells, NHBE) are isolated from the lung epithelium above the bifurcation of the lungs (Lonza, 2025). PBECs can be cultured at the ALI using a defined medium to induce differentiation. Upon differentiation, the PBECs become pseudostratified and polarized and the culture contains ciliated, goblet, and basal cells (Stewart et al., 2012). The TEER and tight junction formation can vary between experiments, the number of times they have been expanded (maximum of three), days of ALI culture and donor. Differentiated PBECs have a high TEER value. The present goblet cells produce mucus. As reviewed by Dong and Zhuang (2024), activity of CYP enzymes is reduced in PBECs compared to the *in vivo* situation and the expression pattern is slightly modified. UDP-glucuronosyltransferases (UGT) can be stimulated with beclomethasone (Kuzuya et al., 2004).

EpiAirway (MaTek Corporation, Ashland, MA) is marketed as mucociliary tissue model composed of multiple layers of PBECs. These can be obtained from either healthy donors or patients with respiratory diseases such as chronic obstructive pulmonary disease (COPD), asthma, cystic fibrosis (CF), smoking history, or allergic rhinitis. EpiAirway tissue is cultured on a microporous membrane and consists of a ciliated apical surface and mucociliary epithelium that produces mucus, contains functional tight junctions, and exhibits beating cilia (MATTEK, 2025, Barilli et al., 2020). The model is marketed as easy to handle. EpiAirway may be maintained at ALI for at least 3 months after shipment by the manufacturer, and the basal side of the insert is maintained with cell culture medium also provided by the manufacturer. EpiAirway has been shown to express the epithelial marker keratin 5 and the mucus transporter MUC5AC (MATTEK, 2025, Bolmarcich et al., 2006; Dvorak et al., 2011; Seagrave et al., 2012).

MucilAir™ is derived from the upper airway epithelium (including tracheal, and bronchial regions). The model is commercially available as a fully differentiated, pseudostratified mucociliary epithelium (EPITHELIX, 2025). This culture consists of the main epithelial cell types found in the human airway, namely, basal progenitor cells, goblet cells, and ciliated cells. It exhibits functional characteristics of the *in vivo* epithelium, including a well-established barrier function supported by tight junctions, which can be assessed by measuring TEER. The model also demonstrates mucus production through goblet cell activity and maintains active mucociliary clearance, an essential defense mechanism of the respiratory tract. MucilAir™ has a long shelf life, remaining functional for several months and up to a year, is produced under GIVIMP-certified conditions since 2023, and is widely used in research, covering applications such as viral infections, inhalation toxicology, and drug delivery. Gene profiling of genes involved in xenobiotic metabolism demonstrated strong similarities with the normal human lung and did not reveal any consistent changes when assessed over a 6-month period (Baxter et al., 2015). Inducibility and activity of CYP1A1/1B1 and activity of CYP2A6/2A13 were present at 1 month in culture and maintained in one tested MucilAir™ donor for several months.

#### 4.1.2 Expression of transporters

P-glycoprotein (P-gp) is an ATP-dependent efflux pump. Previous attempts to characterize P-gp expression and function in the respiratory epithelium have been conflicting. PBECs at day 21 in culture show minimal polarized P-gp mediated efflux of digoxin, but this was not found after 14 days in culture (Madlova et al., 2009). Multidrug resistance-associated protein (MRP)1 and MRP2 expression has also been detected in PBECs (Kuzuya et al., 2004).

On the contrary, functional P-gp expression was not observed in EpiAirway, but MRP1 and breast cancer resistance protein (BCRP) expression are detected in EpiAirway cells (Rotoli et al., 2020). EpiAirway also expresses organic cation transporters (OCTs) at the basolateral side. At the apical side, the most abundant transporter is ATB^0,+^ (*SLC6A14*), that can potentially list among organic cation transporters responsible for drug delivery in the lung (Barilli et al., 2020; Rotoli et al., 2020).

P-gp is functionally present and capable of effluxing in MucilAir. Additionally, BCRP was shown to be functionally present. P-gp, MRP1/2, and BCRP are all expressed on the cell membranes of MucilAir. Ultimately, understanding these differences is essential for accurately predicting uptake of inhaled toxicants.

#### 4.1.3 Primary bronchial cells for uptake assessment

Both EpiAirway™ and MucilAir™ are used for uptake and permeability assessment at the ALI. In side-by-side studies, these mucociliary airway tissues have been used to quantify apparent permeability (P_app_) of model drugs across the epithelium and to compare their barrier properties and predictive value for *in vivo* (nasal) absorption, alongside standard monolayer systems such as Caco-2, Calu-3 and MDCK (Furubayashi et al., 2020). Beyond small-molecule permeability, MucilAir™ has been applied to study the absorption of constituents such as nicotine and ethyl maltol from e-cigarette vapor, underlining its relevance for quantitative respiratory uptake assessment (Reus et al., 2014; Staal et al., 2024b).

The main advantages of these models are their highly relevant physiology, including intact barrier properties, mucus production, ciliary activity, ion transport, and metabolic functions. As the cells are obtained from individuals, there is an inherent variation between donors, although pooled donor cells can be used to reduce this effect. Variation between donors could be considered a disadvantage, as cells from different donors could respond differently to exposures. On the other hand, this variation in cultures also represents the variation in the human population, thereby providing an opportunity for these cells to capture the variation in the population. Culturing of PBECs is relatively costly, as consumables are more expensive than those for other ALI-cultured cells and the models require a relatively long culturing time before they can be used.

### 4.2 Calu-3

#### 4.2.1 ALI culture

Calu-3 cells are a human bronchial lung adenocarcinoma-derived epithelial cell line, widely used in permeability studies due to their ability to form a polarized epithelial monolayer with well-developed tight junctions, desmosomes, and *zonulae adherens,* resulting in high TEER. This makes Calu-3 cells a relevant *in vitro* model that closely mimics the barrier properties of the human airway epithelium (Sibinovska et al., 2020). These cells can be cultured under ALI or LLI conditions, with TEER values ranging from 300 to 800 Ω cm^2^ (ALI) to 600–2500 Ω cm^2^ (LLI) (Zhu et al., 2010; Enlo-Scott et al., 2021). Furthermore, Calu-3 ALI cultivation promotes pseudostratified columnar epithelium with microvilli and increased mucus secretion, expression of transporters and metabolic enzymes, as well as more pronounced tight junction formation (Grainger et al., 2006; Lacroix et al., 2018; Sonntag et al., 2022). Only a few studies have compared the permeability predicted using Calu-3 cells culture in ALI and LLI with *in vivo* observed permeability and when it is the case, the results are similar whatever the culture model (Mathias et al., 2002; Grainger et al., 2006). Co-cultures of Calu-3 cells with macrophages (THP-1) can enhance the physiological relevance of the model especially for particles and aerosols, while they drastically increase the complexity of the model culture and may change the barrier properties (He et al., 2021).

This cell line has low or no CYP metabolic activity (Florea et al., 2001), but their expression can be induced by exposure to certain chemicals, such as polycyclic aromatic hydrocarbons. Therefore, they do not fully replicate the metabolic activity of the human lung *in vivo* (Hukkanen et al., 2002). Regarding cytochrome P450 (CYP) enzymes, documented expression includes CYP1A1, CYP1B1, and CYP3A4/5, although the expression levels are generally lower than those found in liver cells or primary lung tissue and should be experimentally evaluated for each tested compound. Calu-3 cells also express other phase I and phase II metabolic enzymes, such as UDP-glucuronosyltransferases (UGTs) and glutathione S-transferases (GSTs).

#### 4.2.2 Expression of transporters

Calu-3 expresses several uptake and efflux transporters relevant to chemical absorption in airway epithelium such as MRP1, P-gp, the organic cation transporter 3 (OCT3), the organic cation transporter novel type 2 (OCTN2), and the sodium- and chloride-dependent neutral and basic amino acid transporter B (0+) (ATB^o,+^) but not BCRP, with expressions varying with culture mode and duration (Mukherjee et al., 2012; Rotoli et al., 2020). The expression of BCRP in this cell line remains controversial, as some studies report detectable levels while others find none. The efflux transporters MRP1 and BCRP in Calu-3 cells have been found at levels comparable to those observed in PBECs (Sakamoto et al., 2015). In addition, functional studies confirmed apical localization of P-gp transporters. P-gp expression is consistent under LLI and ALI culture conditions (Sibinovska et al., 2020).

#### 4.2.3 Calu-3 for uptake assessment

Calu-3 cells are widely used to study the permeation of drugs and nanoparticles across the airway epithelium (Borchard et al., 2002; Mathias et al., 2002; Bosquillon et al., 2017; Furubayashi et al., 2020; Inoue et al., 2020; Sibinovska et al., 2020). In addition, it serves as a model for investigating airway epithelial barrier integrity, drug transporter expression, and tight junction dynamics in response to environmental stressors such as cigarette smoke (Petpiroon et al., 2023). Moreover, it serves as a relevant model for studying active ion transport, given its high expression of CFTR, and for exploring cellular processes such as endocytosis (Enlo-Scott et al., 2021). A correlation between Calu-3 permeability and rat *in vivo* lung absorption has been reported for a range of small molecules, indicating that Calu-3 can be a useful predictive model for airway absorption (Mathias et al., 2002). The mucus barrier produced in ALI could reach up to 10–20 µm thickness therefore limiting the permeability to lipophilic or high molecular weight compounds (Sonntag et al., 2022). Overall, Calu-3 represents a robust model for deriving epithelial permeability P_app_ and mechanistic transport parameters, while metabolic clearance and complex immune interactions typically require complementary systems or model-based extrapolation.

### 4.3 HBEC2-KT, HBEC3-KT, HBEC12-KT

#### 4.3.1 ALI culture

The human bronchial epithelial cell lines HBEC3-KT, HBEC2-KT, and HBEC12-KT are hTERT- and CDK4-immortalized epithelial cells derived from the bronchial tissue of three donors (Ledo et al., 2023).

Among these, HBEC3-KT cells have been shown to retain characteristics of multipotent stem cells of the lung when cultivated at an air–liquid interface (ALI) for up to twenty-eight days (Vaughan et al., 2006; Delgado et al., 2011).

Delgado et al. demonstrated that when cultured on Matrigel™, or in the presence of fibroblasts, HBEC3-KT cells can also differentiate into Clara cells, columnar ciliated epithelial cells, and goblet cells capable of mucus production. The pluripotency of these cells appears to depend on the microenvironment in which they are cultivated. After several days in ALI culture, they express p63, Keratin 14, MUC5AC, and AQP5 (Delgado et al., 2011; Vaughan et al., 2006). These findings indicate that immortalized HBECs retain the capacity to differentiate into the three major airway epithelial cell types: basal, mucin-producing, and columnar ciliated epithelial cells.

It has also been shown that under certain conditions they can form cyst-like structures resembling lamellar bodies characteristic of type II pneumocytes. Consistent with this, HBEC3-KT cells are positive for surfactant proteins, similar to type II pneumocytes (Delgado et al., 2011). Pluripotent characteristics were also found in HBEC-2KT and HBEC-12KT.

These HBECs retain markers characteristic of several epithelial cell types of the adult lung when tested experimentally in cell culture. This model therefore exhibits many features of adult human lung tissue and could be valuable for understanding not only mechanisms specific to lung cell lineage differentiation but also the pathogenesis of human lung diseases, including lung cancer.

Although these cells maintain many features of human bronchial tissue, few studies have addressed the functionality of these biomarkers. To this end, STAMI is developing a differentiated bronchial model using HBEC-3KT and characterizing it to investigate its structural and functional properties.

The HBEC3-KT, HBEC2-KT, and HBEC12-KT cell lines have been used in carcinogenesis studies to study their abilities to undergo epithelial-mesenchymal cells (EMT) transition and invasion properties (Uppstad et al., 2010; Bersaas et al., 2016; Hýžďalová et al., 2024). An *in vitro* transformation model of human bronchial epithelial cells (HBECs-2 KT and 12 KT) has been used to study long-term effects of tobacco smoke and benzo(*a*)pyrene (BaP) (Bersaas et al., 2016). HBECs that were transformed into EMT showed an upregulation of markers like SNAI1, ZEB1, VIM and MMP2 and a downregulation of FOXA1 and FOXA2, which are typical markers of epithelial-mesenchymal cells (Bersaas et al., 2016). Similarly, HBEC3-KT cells transformed after long term exposure to diesel exhaust particles also underwent EMT (Rynning et al., 2018).

After chronic exposure to BaP and activation of the aryl hydrocarbon receptor (AhR), HBECs also underwent morphological changes accompanied by increased expression of proinflammatory cytokines, such as CXCL5 (Hýžďalová et al., 2024). These cells have been shown to bioactivate BaP through the cytochromes CYP1A1 and CYP1B1, which contribute significantly to the formation of the B [a]P-cis- and trans-7,8-dihydrodiol isomers involved in the carcinogenic process (Uppstad et al., 2010; Ledo et al., 2023).

#### 4.3.2 Expression of transporters

Among transporters, HBECK-3KT cells show high expression of ANT1 and ANT2, which are both members of the adenine nucleotide translocase family located in the mitochondrial membrane. Dysregulation of these transporters has been linked to chronic obstructive pulmonary disease (COPD) (Kliment et al., 2021).

#### 4.3.3 HBEC2-KT, HBEC3-KT, HBEC12-KT for uptake assessment

STAMI is developing a bronchial model using HBEC-3KT cells and is differentiating and characterizing this model to study its structural and functional properties. Although this cell model is relatively new, it shows characteristics that are important for uptake assessment, such as a well-developed barrier and human-relevant expression of transporters. These models represent an intermediate level of biological complexity between immortalized monocultures and primary airway tissues. They are particularly suitable for deriving epithelial permeability coefficients and related transfer rate parameters under differentiated conditions, while also enabling exploration of donor-dependent variability. However, quantitative characterization of pulmonary metabolic clearance and immune-mediated processes generally requires complementary systems or model-based extrapolation.

## 5 Alveolar models

### 5.1 hAELVi

#### 5.1.1 ALI culture

hAELVi (human alveolar epithelial lentivirus-immortalized) is a cell line derived from primary type I alveolar cells (AT1 cells) that has been immortalized by the introduction of a lentivirus. These models are intended to represent the alveolar epithelium in the alveolar region. It is estimated that AT1 cells, which are modelled on hAELVi cells, account for approximately 95% of the alveolar surface area, thus making hAELVi a good proxy to mimic the alveolar barrier (Kuehn et al., 2016).

hAELVi cells can be cultured under both liquid-liquid (LLI) and air-liquid interface (ALI) conditions. When transitioning to ALI, the apical medium is removed, and the cells are fed from the basolateral side. Additional cells could be added to complete the model, for instance a co-culture of hAELVi and NCI-H441 has been successfully cultivated and maintained for a period of at least 30 days (Brookes et al., 2021).

hAELVi cells are characterized by the formation of tight intercellular junctions, often exceeding 1000 Ω cm^2^ and even reaching 2000 Ω cm^2^ or higher in ALI culture (manufacturer data). The obtained barrier demonstrates a low permeability to the hydrophilic markers sodium fluorescein or mannitol. The barrier function is supported by the presence of tight junctions (ZO-1, occludin) and desmosomes (Kuehn et al., 2016; Visigalli et al., 2022). hAELVi cells may form microvilli and lamellar structures and are able to produce a glycocalyx when cultured in ALI conditions (Brandt et al., 2020).

The barrier function of hAELVi cells can be modulated by pro-inflammatory cytokines such as TNF-α and IFN-γ, leading to decrease TEER and increase permeability, thereby mimicking an inflammatory response.

This model is more recent compared to the other cell models, and therefore, specifically for this cell model further research is required to fully characterize the expression of metabolic enzymes and transporters. Another drawback of this cell line compared with the more routinely used model (e.g., Calu-3), is its higher cost and the requirement for specialized culture media and coating solutions (Enlo-Scott et al., 2021).

#### 5.1.2 Expression of transporters

Visigalli, Rotoli et al. (2022) investigated the expression of ABC transporters, on EpiAlveolar™ and hAELVi cells including BCRP (*ABCG2*), P-gP (*ABCB1*); MRP1 (*ABCC1*) in ALI culture and none of these transporters were found to be expressed on the apical membrane of the hAELVI. MRP1 was the only transporter clearly detected and functional on the basolateral side, suggesting that other (unidentified) transporters may be expressed on the apical side.

#### 5.1.3 hAELVi for uptake assessment

The hAELVi cell line is considered to provide a more physiologically relevant representation of the human alveolar epithelium than cell lines such as A549, due to their ability to form a tight barrier and express key markers of alveolar cells. The hAELVi models are valuable for studying both the absorption and inflammatory supporting both submerged and ALI exposures. However, common ABC transporters are not expressed apically in hAELVi models and further information on transporters is missing. Information on the metabolic capacity of these cells is needed to understand if they can metabolize the tested chemicals, which would affect uptake and toxicity assessments. Until these gaps are addressed, uptake studies in hAELVi should be made cautiously, ideally in combination with complementary models and appropriate controls.

### 5.2 hiPSC-based alveolar model

#### 5.2.1 ALI culture

The hiPSC-based alveolar model is based on human induced pluripotent stem cell (hiPSC)–derived alveolar epithelial like cells Type 2 (AEC2). The hiPSC cell line is obtained from the European Bank for induced pluripotent stem cells (EBiSC, 2025) and registered on the Human pluripotent stem cell registry (hPDCreg, 2025). For the differentiation of lung-specific cell types from hiPSCs, key developmental pathways have to be reproduced *in vitro*. This process known as directed differentiation, is typically achieved by a stepwise addition of signaling factors needed for the recapitulation of the embryonic lung development. The protocol that will be used in this project is described in detail by Müller et al., 2023. It entails a series of steps, starting with generating definitive endoderm (DE) and directing it toward anterior foregut endoderm (AFE), followed by the induction of lung progenitor lineages, which finally undergo cell-type specific differentiation and maturation (details are provided in Supplementary Data Sheet 1).

Alveolar epithelial cells type 2 (AEC2) are cuboidal, twice as frequent as AEC1, and specialized in the production and secretion of pulmonary surfactant, that decreases surface tension during breathing. Alveolar epithelial cells type 1 (AEC1) are squamous, flat, terminally differentiated cells that cover the majority of the alveolar area, providing the surface needed for gas exchange and participating in ion and protein transport. They are obtained from AEC2 through a further differentiation step. Only a few hiPSC-based ALI models have been described in the literature to date (Bluhmki et al., 2020; Schruf et al., 2020; van Riet et al., 2020; Bluhmki et al., 2021; Abo et al., 2022; Masui et al., 2022; Moreira et al., 2022; Werder et al., 2022; Hristozov et al., 2024). Some of these models focus on tracheobronchial cells and are therefore based on epithelial cells of the ciliated epithelium or basal cells (Ahmed et al., 2022; Berical et al., 2022; Djidrovski et al., 2023). Although a method for differentiating AEC1 (alveolar epithelial cells type 1) from AEC2 (alveolar epithelial cells type 2) was published by Ghaedi et al. (2014), but without any application in toxicity studies or for disease modeling. Since to date, there has been no widespread application of hiPSC-based alveolar ALI models in safety assessment of chemicals and also not in the field of drug development or disease studies. For ALI *in vitro* models multiple studies use immortalized tumor cell lines, as, e.g., A549. Only few studies are published describing hiPSC-based AEC2 at ALI (Abo et al., 2020; Hekman et al., 2020; Huang et al., 2020; Werder et al., 2022).

As hiPSC are differentiated in AEC2-like cells, the barrier function is lower, compared to bronchial cells, but less data is available. Also, less information is available on the expression of metabolism genes in hiPSC-based alveolar models.

#### 5.2.2 Expression of transporters

In hiPSC-derived alveolar type II (ATII) cells, transporter expression varies with differentiation protocol, but the most consistently reported transporters are (1) ABCA3 (the lamellar body lipid transporter; essential for ATII function and crucial for surfactant phospholipid transport) and (2) The surfactant protein C (SFTPC). ABCA3 is found on the lamellar bodies of AEC2 cells. Studies using hiPSC-AEC2s have shown it can be used to investigate its role in surfactant transport and in diseases caused by ABCA3 mutations (Sun et al., 2021). SFTPC is processed within the cell, and its mislocalization can be studied in hiPSC-AEC2 models of lung disease. This allows researchers to investigate how mutations in SFTPC affect its processing and the cell’s response (Alysandratos et al., 2021).

Recently, it has been revealed that SARS-CoV-2 infection depends on the host cell factors ACE2 and TMPRSS2 (Hoffmann et al., 2020). AEC2 have been identified as the cell type, which expresses both factors in the distal lung. Spike proteins of SARSCoV-2 bind to ACE2 as an entry receptor on the target cell surface and utilize TMPRSS2 for the spike protein priming, which allows fusion of viral and cellular membranes (Shang et al., 2020). The membrane receptors targeted by SARS-CoV-2, angiotensin converting enzyme 2 (ACE2) and transmembrane serine protease 2 (TMPRSS2), have been identified to be expressed by alveolar epithelial cells type 2 (Chandrasekaran and Fernandes, 2020). Additionally, CFTR, which plays also a role in the pathogenesis of CF (Krencik et al., 2011), has been determined on the surface of differentiated AEC2 (Müller et al., 2023).

#### 5.2.3 hiPSC-based alveolar models for uptake assessment

The hiPSC-based based alveolar model that will be used in this project is an ALI model of hiPSC-derived alveolar like cells type 2. The model is considered to provide a more physiologically human-relevant simulation of the human alveolar epithelium than cell lines such as Calu-3, due to their cell source. Depending on the donor (healthy donor or donor with specific disease) of the hiPSC cell line used for the differentiation, besides healthy models, the *in vitro* model could be adapted patient-specific/disease-specific (personalized *in vitro* model). This model allows for detailed mechanistic studies, but also uptake and permeability studies at the ALI.

### 5.3 ALIsens model

#### 5.3.1 ALI culture

The ALIsens model is aco-culture alveolar model developed by LIST, combining three different cell lines grown on 6-well cell culture inserts. The system is seeded on the membrane of the inserts, allowing the apical side to grow at the ALI while maintaining the basolateral side under submerged conditions (Chary et al., 2019). The model consists of EAhy.926 endothelial cells and THP-1–derived dendritic-like cells in the basolateral compartment, cultivated under submerged conditions, whereas the apical side, comprising A549 alveolar epithelial cells and THP-1 derived macrophage-like cells, is maintained at the ALI. The 3 cell lines included in the model are cultivated, differentiated, and seeded following the protocol developed at LIST (Supplementary Figure S1). The origin and characteristics of the cell lines used in the model are summarized in Supplementary Data Sheet 1.

#### 5.3.2 Expression of transporters

Although no published study has yet directly characterized transporter expression or function within the ALIsens models, studies performed on the individual cell lines composing the model, namely, A549 alveolar epithelial cells and EA.hy926 endothelial cells, can serve as a mechanistic reference framework. A549 cells demonstrate functional expression of P-gp, as shown in studies linking P-gp levels to intracellular dexamethasone concentrations (Cohen et al., 2010). Additional work reviewing ABC transporters across pulmonary epithelial *in vitro* models confirms that members of the ABC family (e.g., P-gp, MRP1, BCRP) can be active under certain culture conditions, though expression patterns vary substantially depending on cell type and culture parameters (Simon et al., 2025). These data suggest that A549 within ALIsens may support efflux-mediated regulation of intracellular xenobiotic concentrations.

On the endothelial side, EA.hy926 cells express the cationic amino-acid transporter CAT 1 (SLC7A1), with demonstrated regulation of its mRNA by protein kinase C (PKC) activation (Gräf et al., 2001). This transporter likely contributes to regulated uptake of endogenous substrates (e.g., L-arginine), influencing endothelial signaling (e.g., NO production) and potentially modulating the barrier.

#### 5.3.3 ALIsens model for respiratory uptake

The ALIsens model has primarily been applied to the identification of chemical respiratory sensitizers, demonstrating its capacity to discriminate between respiratory sensitizers, irritants, and skin sensitizers (Chary et al., 2019; Burla et al., 2025). More recently, it has been used to evaluate inflammatory and immunomodulatory responses following exposure to advanced nanomaterials. Therefore, the model is currently best suited for immune-related and inflammatory endpoints.

A limitation of the current model is that A549 cells may not form fully functional tight junctions, which affects barrier integrity and controlled uptake assessment. In addition, further research is required to fully characterize the expression of metabolic enzymes and transporters. This limits their use for comprehensive uptake and toxicity assessments.

## 6 Selection of a cell model for respiratory uptake assessment

All models described form a barrier and can be cultured at the ALI. Assessment of uptake using both bronchial and alveolar models can be relevant for potential differences in uptake between locations in the respiratory tract. Additionally, some models have been shown to be able to produce mucus or surfactant, whereas for others this has not been established. This provides the potential to assess the potential role of mucus or surfactant in respiratory uptake.

For many cell models, little information is available on the expression of metabolic enzymes or transporters on the cell membrane. To understand potential differences in the outcome and to use any of these cell models for human relevant uptake assessment, it is necessary to characterize transporter expression (Mercier et al., 2019) and evaluate the expression of metabolic enzymes (Baxter et al., 2015). Transporter expression data are only interpretable in terms of functional impact on uptake/efflux when the exposure conditions: delivered cellular dose, apical contact time, and physicochemical state of the test substance, are quantitatively defined. Indeed, the ALI-culture allows the use of different exposure methods, which can be selected based on the substance properties. Different exposure methods may also result in differences in exposure kinetics, which makes the appropriate exposure methods essential for uptake studies. Within this project, the exposure methods are harmonized to be able to compare the different cell models under similar controlled conditions. Readouts should include measuring chemical concentrations in the apical wash, within the cells, and in the basolateral medium at multiple time points to calculate apparent permeability (P_app_), uptake percentages, and to enable integration into PBK models.

## 7 Chemicals

### 7.1 Rationale

To assess the suitability of *in vitro* models for respiratory uptake assessment, we will focus on a compound class with a variation in volatility and affinity for protein binding, but also with similarities, which helps in performing RAx. This approach focusses specifically on inhaled chemicals, meaning that particles, as their uptake mechanisms are different from chemical uptake, and reactive substances, as these generally cause local effects, are excluded. We have selected per- and polyfluoroalkyl substances (PFAS) are a large class of synthetic fluorinated organic chemicals that have attracted increasing scientific and regulatory concern due to their extreme chemical stability, environmental persistence, and global distribution (OECD, 2021). The chemical characteristics of the selected PFAS are listed in Table 3. The C-F bond is among the strongest in organic chemistry, conferring resistance to thermal, chemical, and biological degradation. Consequently, PFASs are referred to as “forever chemicals”, and their ubiquitous presence has been reported across multiple environmental compartments including air, water, soil, and biota (Abunada et al., 2020; E. E. A, 2024). PFASs are emitted from different sources and are detected in outdoor and indoor air (D’Ambro et al., 2021; D'Ambro et al., 2023; Li et al., 2024; Minucci et al., 2025). This is not limited to volatile PFASs (e.g., FOSA); also non-volatile PFASs (e.g., PFOS, PFOA, PFNA) can be airborne. Indeed, although PFASs are characterized by low volatility, particularly for long-chain perfluoroalkyl acids such as PFOS and PFOA, they are nonetheless widely detected in the atmosphere across urban, coastal, and even remote regions such as the Arctic (MacInnis et al., 2019). This apparent paradox may be explained by several mechanisms. First, their precursors such as fluorotelomer alcohols (FTOHs), perfluoroalkyl sulfonamides (FASAs), and sulfonamide ethanols (FASEs), are semi-volatile and can readily enter the gas phase. These precursors undergo atmospheric oxidation to form persistent PFAAs that subsequently deposit to surfaces or participate in long-range transport. Second, PFASs and ionic species can adsorb onto aerosols and dust particles, allowing them to be transported over long distances despite their low vapor pressure (Yang et al., 2025) Table 3.

**TABLE 3:** Selected PFAS and their (physical-chemical) properties.

Abbreviation	PFNA	FOSA	PFOS	PFOA	PFBS	PFPeA
CAS no.	375-95-1	754-91-6	1763-23-1	335-67-1	375-73-5	2706-90-3
Full name	Perfluorononaoic acid	Perfluorooctane sulfonamide	Perfluorooctane sulfonic acid	Perfluorooctanoic acid	Perfluorobutane sulfonic acid	Perfluoropentanoic acid
PFAS subgroup	Long-chain PFCAs	FASAs	Long-chain PFSAs	Long-chain PFCAs	Short-chain PFSAs	Short-chain PFCAs
Molecular structure	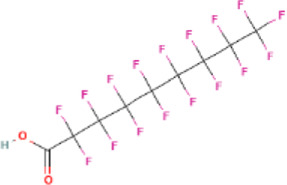	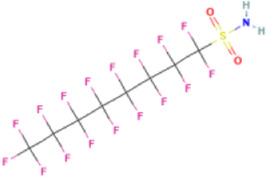	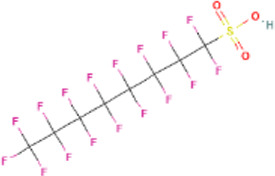	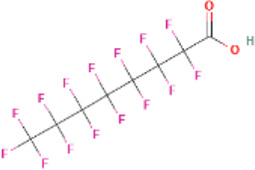	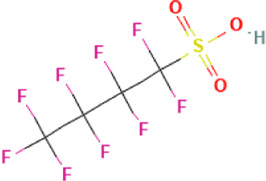	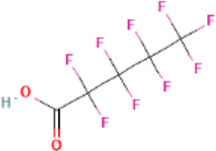
Molecular weight	464.08 g/mol	499.15 g/mol	500.13 g/mol	414.07 g/mol	300.10 g/mol	264.05 g/mol
Vapour pressure	0.08 mmHg	2.5 × 10^−4^ mmHg	2.48 × 10^−6^ mmHg	0.15 mmHg	0.027 mmHg	∼0.001–0.003 mmHg
pKa	−0.21	∼6–8	<1.0	−0.5–4.2	−3.31	∼–0.1
logKow	∼4.96	−3.0–4.0	not measurable	6.3	−1.3	3.8–4.0
Solubility in water	9 mg/L at 25 °C	0.5 mg/L	680 mg/L	3300 mg/L	510 mg/L	14 g/L at 20 °C
Melting point	68 °C–70 °C	35 °C–37 °C	No data	54.3 °C	20 °C–24 °C	28 °C–30 °C
Boiling point	>250 °C	>200 °C	249 °C	192 °C	210 °C–212 °C	185 °C–200 °C
Quick note	Indoor dust/sea-spray relevant	Volatile PFOS precursor; gas + particle phases. Very weak acid/behaves neutrally	Global pollutant; long human half-life	Widely studied; strong protein/transporter interactions, when heated to decomposition, it emits toxic vapours	Shorter half-life	​
Rationale	Metabolite of volatile FTCA, long biological half-lives, found in indoor air, evidenced toxicity	Sulfonamide (neutral precursor), semi volatile, detected in indoor and outdoor air	Legacy chemical, abundant atmospheric occurrence, evidenced toxicity	Legacy chemical, abundant atmospheric occurrence, evidenced toxicity	Short-chain, PFOS replacement, present in indoor air/dust	Distributed in both the gas phase and the particle phase, immune-related responses in mice
Pubchem	Perfluorononanoic acid | C8F17COOH | CID 67821 - PubChem	Perfluorooctanesulfonamide | C8H2F17NO2S | CID 69785 - PubChem	Perfluorooctanesulfonic acid | C8F17SO3H | CID 74483 - PubChem	Perfluorooctanoic acid | C8HF15O2 | CID 9554 - PubChem	Perfluorobutanesulfonic acid | C4F9SO3H | CID 67815 - PubChem	Perfluoropentanoic acid | C4F9COOH | CID 75921 - PubChem

This will result in human exposure to PFAS via inhalation - something that is relatively important to the microenvironment of the PFAS speciation (Christensen and Calkins, 2023). Therefore, despite their low volatility, the presence of PFAS in air makes them a highly relevant chemical class for evaluating lung permeability *in vitro*. PFASs were prioritized based on their established toxicological profiles and to represent main PFAS chemical subgroups (based on their functional group).

### 7.2 Uptake/efflux mechanisms

Because PFAS exist almost entirely in their anionic forms at physiological pH, they rely predominantly on carrier-mediated transport rather than passive diffusion. PFAS can enter cells via active transport by various solute carriers (*SLC*) and leave cells using ATP-binding cassette (*ABC*) transporters (Collier and Lavado, 2024). Organic anion transporters (OAT) 1, OAT3 and OAT4 (*SLC22A6*, *A8* and *A11*) are involved in uptake and excretion of PFAS in renal cells (Louisse et al., 2023; Louisse et al., 2024). Among the perfluoroalkyl carboxylic acids (PFCAs) and perfluoroalkyl sulfonic acids (PFSAs) assessed were some overlapping with our selection (namely, PFOA, PFNA, PFBS, and PFOS). OAT1 transport plays a role for PFOA and OAT3 for PFOA and PFNA. The organic-anion-transporting polypeptide family (OATPs) has also demonstrated transport activity for PFAS. Hepatic uptake of PFHxS, PFOS and to a lesser extent PFBS are mediated by OATP1B1, 1B3 and 2B1 (*SLCO1B1*, *1B3* and *2B1)* (Zhao et al., 2016). Similar results were obtained with caco-2 cells, confirming the role of OATPs in the uptake of PFAS substances (Kimura et al., 2017). In the liver, the Na + -taurocholate co-transporting polypeptide (NTCP, *SLC10A1*) was demonstrated to transport PFBS, PFHxS, and PFOS (Zhao et al., 2015). The fatty acid transporter CD36 was also found to play a role in cellular uptake of PFAS in retinal epithelial cells. The organic solute transporters (OST) α/β (*SLC51A* and *B*), the apical sodium bile acid transporter (ASBT,*SLC10A2*) and urate transporter 1 (URAT1, *SLC22A12*) are also able to uptake PFAS (Yang et al., 2010; Zhao et al., 2015; Camdzic et al., 2022).

Efflux mechanisms remain less well characterized but other transporters, such as MRP2 (*ABCC2*) and BCRP (*ABCG2*) may be relevant in placenta tissue (Solan et al., 2023). PFOA is a substrate of BCRP (Dankers et al., 2013).

PFAS can also modulate transporter activity, for example, by inhibiting P-gp (*ABCB1*) (Dankers et al., 2013; Tastet et al., 2023) or by impacting the expression of other transporters (Lim et al., 2022).

No specific data are available on transporter-mediated uptake of PFAS in the respiratory epithelium. However, it can be assumed that transporters identified in other tissues and in the lung may play a role. In the healthy lungs, OAT family members are generally not expressed, with the exception of OAT2 for which PFAS are not known substrates (Cropp et al., 2008; Dankers et al., 2013; Lim et al., 2022). Among previous transporters OATP2B1, MRP1,2,4-6 and BCRP are in the respiratory tract and may contribute to PFAS transport across airway epithelia (Bleasby et al., 2006; Chen et al., 2024; Dong and Zhuang, 2024).

### 7.3 Chemical analysis

PFAS in cell lysates and exposure media will be quantified with LC-MS/MS. Procedures are based on established procedures for biological and environmental samples and are going to be adapted accordingly (CDC, 2020). Methodological parameters (e.g., extraction efficiency, LOD/LOQ) will be determined for each matrix, including cell culture medium, cellular material, and sample preparation plastics when relevant.

A general analytical approach will include protein-precipitation–based extraction using LC-MS grade solvents and isotopically labelled internal standards for the target PFAS to support recovery correction. The chromatographic separation of the compounds of interest will be performed with reversed-phase LC and a PFAS-delay setup to minimize system background. Detection will be carried out on triple-quadrupole MS instruments operating in ESI^−^ with compound-specific MRM transitions. Calibration will cover a broad dynamic range, whereas LOD and LOQ will be reported as determined for each analytical sequence and depending on instrument performance.

Sample preparation will be performed by adding methanol containing the internal-standard mixture, followed by vortexing, centrifugation, and transfer of the supernatant into PFAS-free vials. All consumables that are going to be used should be PFAS-free (polypropylene/HDPE or PEEK components) to minimize background contamination.

UHPLC analysis will be performed using organic solvents (methanol or acetonitrile) as mobile phases with a gradient elution and MS settings that are going to be optimized according to instrument specifications. Typical sensitivity for this method achieves low-ng/L detection; however, matrix-specific LOD/LOQ values will be experimentally established for each series of samples, taking into account reagent blank levels, instrument performance, and recovery of fortified quality control samples.

Finally, all analytical steps will be adapted as required for new sample types used, taking into account matrix-specific characteristics, expected PFAS concentrations, and potential interferences arising from different cell models or exposure setups. Available analytical guidance documents on PFAS analysis (e.g., from the EURL POPs (2024)), will be taken into account as far as they are applicable during method set-up and characterization.

## 8 *In silico* approaches to fill data gaps

QSPR and QSAR are statistical modelling approaches aiming at predicting physicochemical properties, or biological, pharmacological, toxicological and ADME properties respectively, based on molecular structures which could be represented as Simplified Molecular Input Line Entry System (SMILES) strings. Traditional steps involve (i) splitting of experimental data into training and test se; (ii) application of a statistical algorithm on the training set; (ii) evaluation of its performance on the test set and (iv) prediction of target compounds and evaluation of their reliability via applicability domain metrics. QSAR/QSPR models are more prone to output reliable predictions when they are based on extensive data availability. This may not apply in the opposite case, where RAx, a data gap filling methodology, may instead be more suitable. RAx aims at filling toxicological knowledge gaps by analogy between compounds which value for the property of interest has been experimentally established (“source” compounds) and compounds whose value is unknown and is to be predicted (“target” compounds) (Lizarraga et al., 2023; Roy and Banerjee, 2024). Similarities with respect to structural, physicochemical, toxicokinetic features may drive RAx. Automated workflows are now available and can support the expert judgement in evaluating the scientific validity of a RAx (OECD, 2026).

Several frameworks were developed in the latest years to guide risk assessors and regulators through a consistent evaluation of evidence generated via the mentioned *in silico* tools and to ultimately increase confidence in them. The latest ones were the *(Q)SAR Assessment Framework* (QAF) developed by OECD (Gissi et al., 2024) and *Read*-*Across* Assessment Framework (RAAF) and *Guidance on the use of read-across for chemical safety assessment in food and feed* by ECHA and EFSA respectively.

In this project, due to the specific physicochemical properties of PFAS, resulting mainly from the presence of a strong C-F bond, predictions of water solubility (logS_w_), vapor pressure (logVP) and octanol-water partition coefficient (logK_ow_) will be conducted using local QSPR models (Leung et al., 2023; Sosnowska et al., 2023; Mudlaff et al., 2024) dedicated for PFAS. Developed models based on the calculated data using COSMO-RS (Conductor-Like Screening Model for Realistic Solvents) algorithm satisfy the OECD’s QAF and are freely deployable online at https://physchempfas.streamlit.app/. Predictions of the physicochemical properties of PFAS can be treated as single values or as supporting data for additional analyses, such as PBK modeling or RAx. Structural and physicochemical similarities between target and source PFAS will underpin the RAx approach, the application of which will be guided by the RAAF. An endpoint which will be modelled via RAx is P_app_, to allow for PBK modelling of non-tested PFAS. P_app_ is a key parameter governing absorption across epithelial cell barriers. This parameter can be determined through *in vitro* assays using the Caco-2 cell line, which differentiates to intestinal enterocyte-alike cells. Experimentally derived or *in silico* predicted P_app_ values from Caco-2 assay are typically used to parametrize both dietary and respiratory absorption in PBK modelling. However, the Caco-2 assay may not be fully representative of absorption across the pulmonary mucosa, compared to the Calu-3 assay (the most used lung cell line, and the one offering most data available in the literature, for example, in terms of expression of P-gp (Selo et al., 2021). In this project, the use of P_app_ obtained via respiratory *in vitro* assays will convey more robust biological relevance to the RAx than otherwise determinable via Caco-2 cells.

## 9 PBK models

Physiologically Based Kinetic (PBK) models provide internal chemical exposure (concentrations over time) across life stages and exposure routes by simulating the absorption, distribution, metabolism, and excretion (ADME) of chemicals in the human body. They enable both forward (external to internal exposure) and reverse (internal to external exposure) dosimetry. PBK models are generally categorized as either generic or substance-specific (Pletz et al., 2020). Generic PBK models are defined by a standardized structure applicable to different substances or substance families and are frequently embedded in risk assessment tools or platforms such as TK-plate or httk (Pearce et al., 2017; Dorne et al., 2023), making them user-friendly. Currently, bottom-up approaches are widely used to parametrize such models, integrating compounds specific *in vitro* derived ADME data. In contrast, substance-specific PBK models incorporate detailed physicochemical and mechanistic properties to predict tissue distribution and metabolite formation. These models are typically more complex, less accessible on standard platforms, and may require longer computational times to obtain the outputs relative to generic models. Recent advances have focused on refining PBK models for PFAS, with an emphasis on vulnerable populations and complex exposure scenarios.

PFAS display atypical toxicokinetics (TK) due to their chemical structure: near-complete ionization at physiological pH, exceptionally strong protein binding, active transporter-mediated distribution/elimination, life-stage transfer (placenta, milk), and very long human half-lives (3.8 years for PFOA and 5.4 years for PFOS) (Pletz et al., 2020). Due to their long elimination of several years, application of a generic PBK model on PFAS exposure can result in substantial underestimation of their internal concentrations. Therefore, over the past 15 years, substance-specific PBK models for legacy PFAS (PFOS, PFOA) and several “short-chain” or alternative chemicals (e.g., PFHxS, PFBS, PFNA/PFDA) have shifted from using empirical elimination constants toward PBK models that explicitly include the transporters and physiological processes (kidney reabsorption, bile cycling, high protein binding) that are responsible for the very long persistence of PFAS in humans (Zhao et al., 2023). East et al. (2023) identified 33 PBK modeling studies for different species including humans, rodents, and monkeys. Among these, the most commonly used PBK model today is the model from Loccisano et al. (2012), Loccisano et al. (2013), particularly used in the EFSA document on PFAS (Chain et al., 2020). This model, developed for monkeys and humans (Loccisano et al., 2011), included at first nine compartments with perfusion-limited for PFOS and PFOA, assuming oral uptake and no metabolism (Loccisano et al., 2011). This model was subsequently extended to a rat model (Loccisano et al., 2012) to include gestational and lactational stages by adding compartments for the mammary gland, placenta, fetus, and milk in humans (Loccisano et al., 2012; Loccisano et al., 2013). Recently another oral PBK model has been published (Deepika et al., 2021).

Fàbrega et al. (2014) introduced an adult PBK model for PFOS and PFOA, that incorporated renal transporter-mediated reabsorption in the filtrate compartment and a saturable reabsorption process. In a subsequent study, Fàbrega et al. (2016) included uncertainty in biochemical parameters to improve predictive performance. Both models were validated using human autopsy data, including PFAS concentrations measured in plasma and various organs (Pérez et al., 2013). This adult PBK model was later extended to other PFAS (Fàbrega et al., 2015): PFBS, PFHxS, PFOS, PFDS, PFHxA, PFHpA, PFNA, PFDA, PFUnDA and PFTeDA. Subsequently, several PBK models were developed using rat data as the basis for extrapolation to humans in order to support health risk assessments. These include models for PFOA (Worley et al., 2017), PFOS (Kamiya et al., 2020), PFNA and PFDA (Kim et al., 2019), and PFHxS (Kim et al., 2018).

For sensitive human population (e.g., children and pregnant women), several PBK models have been published for PFOS and PFOA (Wu et al., 2015; Ruark et al., 2017; Brochot et al., 2019; Chou and Lin, 2019; Rovira et al., 2019; Chou and Lin, 2021; Deepika et al., 2021; Ratier et al., 2024). To date, however, no pregnancy PBK models have been developed for other PFAS.

While most PFAS PBK models have focused on oral and systemic kinetics (drinking water, dietary exposure), inhalation is becoming an increasingly relevant exposure route for occupational exposure, particularly through contaminated dust, and aerosols (de la Torre et al., 2019; Mazumder et al., 2023). The existing models can be adapted to inhalation exposure, including aerosols and volatile precursors (e.g., FTOHs) by adding inhalation absorption submodels (*i.e.*, additional modules with equations describing deposition, dissolution, and transfer of inhaled chemicals across the respiratory epithelium).

Inhalation adds two additional layers of complexity to generic PBK models: i) airway dosimetry prior to systemic entry (gas uptake, particle deposition and dissolution, mucociliary clearance), and ii) ventilation-driven kinetics that make the lung a high-throughput interface rather than a simple tissue compartment (Yeh and Schum, 1980; Louisse et al., 2023; Zhao et al., 2023). These physiological processes inform how alveolar ventilation, blood perfusion, and mucus clearance drive uptake and washout for non-reactive gases or particles.

Physiological parameters, required for PBK modeling, are available for most of the standard species/sex/age (organ volumes, flows, glomerular filtration rate) (Louisse et al., 2023; Zhao et al., 2023). However, current pulmonary PBK compartments often rely on simplified assumptions: instantaneous absorption from alveolar space, or permeability estimates extrapolated from oral absorption or lipophilicity-based QSARs (Louisse et al., 2023; Zhao et al., 2023). These approaches ignore the rate-limiting role of epithelial barriers and the influence of ionization, chain length, and surface activity of PFAS. Consequently, several chemical-specific properties and the actual uptake mechanisms remain poorly predictable and must be informed using experimental data.

Indeed, PFAS display several particularities: strong plasma protein binding, low or no metabolism, tissue accumulation (particularly in the liver), inconstant enterohepatic recycling, and extensive renal tubular reabsorption mediated by OAT, OATPs, NTCP and ASBT that largely determine their long human half-lives (Zhao et al., 2015; Alesio et al., 2022; Niu et al., 2023; Zhao et al., 2023; Vujic et al., 2024; Starnes et al., 2025; Yang et al., 2025). These processes are the main determinants of the long persistence, species differences and non-linearities in internal dosimetry. Of all the developed PBK models considering the protein binding, two studies include the active transport (Worley, Lin), five consider life stages variations (Fàbrega et al., 2014; Chou and Lin, 2019; Deepika et al., 2021; Ratier et al., 2024). These two aspects: protein binding and life-stage physiology address distinct sources of kinetic variability, and have been treated independently in PFAS PBK model development. To date, no PBK model has explored the inhalation route of PFAS *per se* in humans. Only two studies provide kinetic profiles after inhaled exposure to PFAS: Himmelstein et al. (2012) in rats, and Zhu, Pan et al., (Zhu et al., 2023), in mice. The so far cited inhalation models also differ regarding complexity, using one single lung compartment model to more detailed and potentially physiologically more accurate structures. One of the most recently developed inhalation models, PBKiT, divides the human respiratory tract into 25 sub-compartments, which describe the upper to lower respiratory regions. It remains an open question how much complexity is finally needed for accurate PFAS PBK model predictions and which NAM models will be needed to provide relevant compound specific-information in the different regions.

## 10 Next steps

The information on cell models and chemicals will be used to design an experimental plan for respiratory uptake assessment, considering the (physical-chemical) properties of the selected PFAS and the human relevancy of the cell models. RAx will be used to fill data gaps, to ultimately allow PBK modelling of other substances from the same class.

In addition to the properties of the cell model, the choice of cell models will also depend on the expected deposition in the respiratory tract for each chemical (Lippmann et al., 1980). The method to expose the cells (quasi-ALI, nebulization or directed flow or flow past systems) will be based on the physical chemical properties of the substance, while ensuring accurate information on deposited dose over time to determine mass balance for uptake assessment. The selected cell models will be characterized with regard to barrier formation and the expression of relevant enzymes and transporters (using proteomics), to allow assessment of human relevancy of the model. Cytotoxicity measurement will be done prior to transport studies to ensure that the transport experiments are conducted in a sub-toxic dose range. Toxicity assessment will focus on the integrity of the cell barrier and cell viability. Apparent permeability coefficients (P_app_) will be measured. P_app_ coefficients obtained *in vitro* will be used as input parameter for PBK modelling.

Quantitative Structure-Property Relationships (QSPR) models, along with or other *in silico* tools (i.e., read-across to predict kinetic for target chemicals based on similar source compounds), will be used for PBK model parameterisation. This approach will help overcome the limitations in the broader application of PBK models, which are often restricted due to gaps in chemical-specific input data. Additionally, computational techniques, such as molecular docking, will be employed to estimate parameters such as protein binding. By predicting how a compound interacts with proteins, models help in determining their bioavailability and distribution, which are the key factors affecting the transport of chemicals across biological membranes (Bosquillon, 2010).

The NAM data will be used to predict the bioavailability of PFAS compounds in the human organism using PBK modelling and sensitivity analyses will inform about their impact on the model results. A tiered approach will be followed starting with *in silico* prediction to continue to *in vitro* measured data. Different *in vitro* models will be compared to assess their reproducibility and the need for integrating region specific barrier information. In addition, generic assumptions will be tested, e.g., the use of categorial data like high, low or moderate uptake in comparison to compound specific individual data.

Ultimately, the project described in this manuscript will result in a guidance document on the use of respiratory *in vitro* models for uptake assessment. With that, this project will strengthen the regulatory applicability of *in vitro* models and PBK models for assessment of respiratory uptake.
