# Supplementary material for: NAMs for closing knowledge gaps in assessment of respiratory uptake – a PARC project
Source: Front Toxicol. 2026 Jun 3;8:1764276. doi: 10.3389/ftox.2026.1764276 (PMC13286702; doi:10.3389/ftox.2026.1764276)
Supplement: Supplementary file 1 [file DataSheet1.docx]

**Supplementary file 1: Details on the ALIsens model**

Table S1: description of the cell lines used in the ALIsens model

| *In vivo* target / location in the respiratory tract | Cell line | Origin | Characteristic | Metabolism |
| --- | --- | --- | --- | --- |
| Alveolar type II epithelial cells / alveoli | A549 | Human alveolar epithelial adenocarcinoma cell line (Lieber, Smith et al. 1976) | Surfactant production; enables growth and exposure at the ALI | Low metabolism, expression of CYP1A1, CYP1B1, 2B6, 2C, 2E1, 3A5, 3A7 (Hukkanen, Lassila et al. 2000) |
| Endothelial cells / blood vessels (air-blood barrier) | Eahy.926 | Human somatic cell hybrid between umbilical vein cells and A549 cells (Edgell, McDonald et al. 1983) | Expression of adhesion molecules | Nrf2 translocation (that directs metabolic genes) (Klein, Cambier et al. 2017) |
| Dendritic cells / circulating | THP-1 | Human monocytic leukemic cell line (Tsuchiya, Yamabe et al. 1980) | Widely used as a model for human dendritic cells (validated *in vitro* skin sensitization assay)(OECD 2024) |  |
| Macrophages / alveolar lumen | Differentiated THP-1 | Human monocytic leukemic cell line (Tsuchiya, Kobayashi et al. 1982) | Differentiated into macrophage-like phenotype upon PMA stimulation; expresses macrophage markers and phagocytic activity | CYP2C19 expression (Cho, Ahn et al. 2023) |


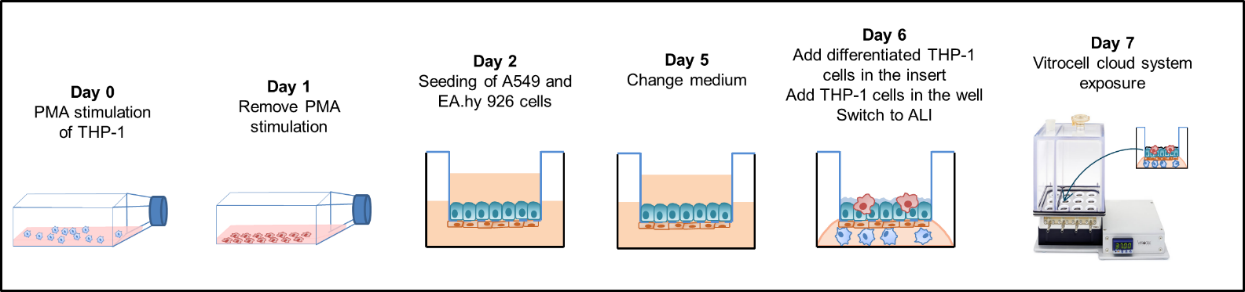


|  |
| --- |

**Figure S1:** Seeding scheme for the coculture system in a 6-well plate format using R5 macrophage-like cells.


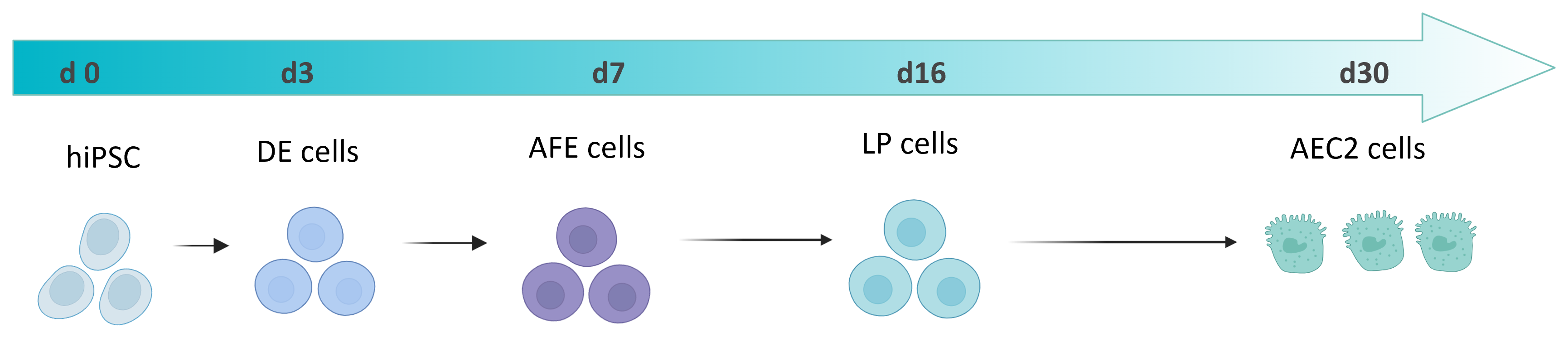


Figure S2: Schematic overview of directed differentiation of hipSC to AEC2-like cells. The AEC2 like cells were generated under 2D-conditions via directed differentiation within 30 days, based on the protocol of Müller et al. 2023 (Müller, Kohl et al. 2023) DE: Definitive Endoderm, AFE: Anterior Foregut Endoderm, LP: Lung Progenitor cells, AEC2: Alveolar-epithelial-like cells type 2. Created with BioRender.com
